# MiT Family Translocation Renal Cell Carcinoma: from the Early Descriptions to the Current Knowledge

**DOI:** 10.3390/cancers11081110

**Published:** 2019-08-03

**Authors:** Anna Caliò, Diego Segala, Enrico Munari, Matteo Brunelli, Guido Martignoni

**Affiliations:** 1 Department of Diagnostic and Public Health, Section of Pathology, University of Verona, Verona 37134, Italy; 2 Department of Pathology, Pederzoli Hospital, Peschiera del Garda 37019, Italy; 3 Department of Pathology, Sacro Cuore Hospital, Negrar 37024, Italy

**Keywords:** MiT family translocation renal cell carcinoma, Xp11 translocation renal cell carcinoma, t(6;11) translocation renal cell carcinoma, FISH, TFE3, TFEB, TFEB-amplified renal cell carcinoma

## Abstract

The new category of MiT family translocation renal cell carcinoma has been included into the World Health Organization (WHO) classification in 2016. The MiT family translocation renal cell carcinoma comprises Xp11 translocation renal cell carcinoma harboring *TFE3* gene fusions and t(6;11) renal cell carcinoma harboring *TFEB* gene fusion. At the beginning, they were recognized in childhood; nevertheless, it has been demonstrated that these neoplasms can occur in adults as well. In the nineties, among Xp11 renal cell carcinoma, *ASPL*, *PRCC*, and *SFPQ* (*PSF*) were the first genes recognized as partners in *TFE3* rearrangement. Recently, many other genes have been identified, and a wide spectrum of morphologies has been described. For this reason, the diagnosis may be challenging based on the histology, and the differential diagnosis includes the most common renal cell neoplasms and pure epithelioid PEComa/epithelioid angiomyolipoma of the kidney. During the last decades, many efforts have been made to identify immunohistochemical markers to reach the right diagnosis. To date, staining for PAX8, cathepsin K, and melanogenesis markers are the most useful identifiers. However, the diagnosis requires the demonstration of the chromosomal rearrangement, and fluorescent in situ hybridization (FISH) is considered the gold standard. The outcome of Xp11 translocation renal cell carcinoma is highly variable, with some patients surviving decades with indolent disease and others dying rapidly of progressive disease. Despite most instances of t(6;11) renal cell carcinoma having an indolent clinical course, a few published cases demonstrate aggressive behavior. Recently, renal cell carcinomas with *TFEB* amplification have been described in connection with t(6;11) renal cell carcinoma. Those tumors appear to be associated with a more aggressive clinical course. For the aggressive cases of MiT family translocation carcinoma, the optimal therapy remains to be determined; however, new target therapies seem to be promising, and the search for predictive markers is mandatory.

## 1. Xp11 Translocation Renal Cell Carcinoma

Xp11 translocation renal cell carcinoma is a distinctive subtype of renal cell carcinoma, characterized by several chromosomal translocations involving the *TFE3* gene, located on chromosome Xp11.2. In these tumors, the *TFE3* transcription factor gene is fused by translocation to one of several other genes [1,2,3,4,5,6,7,8,9]:

- t(X;1) (p11.2;q21.2) gene *PRCC*
- t(X;17) (p11.2;q25) gene *ASPL* (*ASPSCR1*)
- t(X;1) (p11.2;p34) gene *SFPQ* (*PSF*)
- t(X;17) (p11.2;q23) gene *CLTC*
- t(X;3) (p11.2;q21) gene *PARP14*
- t(X;10) (11.2;q23) unknown gene
- t(X;17) (p11.2;q21.33) gene *LUC7L3*
- t(X;19) (p11.2;q13.3) gene *KHSRP*
- t(X;17) (p11.2;p13) gene *DVL2*
- t(X;22) (p11.2;q11.21) gene *MED15*
- t(X;6) (p11.2;q25.3) gene *ARIDB*
- t(X;5) (p11.2;q31.2) gene *MATR3*
- t(X;1) (p11.2;p31.1) gene *FUBP1*
- t(X;11) (p11.2;q13.1) gene *NEAT1*
- t(X;10) (p11.2;q22.2) gene *KAT6B*
- inv (X) (p11.2;q12) gene *NONO* (p54*nrb*)
- inv(X) (p11.2;p11.3) gene *RBM10*
- inv(X) (p11.23;p11.23) il gene *GRIPAP1*

The three most common Xp11 translocation renal cell carcinomas are those bearing the t(X;1) (p11.2;q21) which fuses the *PRCC* and *TFE3* genes, the t(X;17) (p11.2;q25) which fuses the *ASPL* and *TFE3* genes, and the t(X;1) (p11.2;p34) which fuses the *SFPQ (PSF)* and *TFE3* genes [10]. Interestingly, t(X;17) renal cell carcinoma or alveolar soft part sarcoma harbor the same *ASPL-TFE3* fusion gene [11]. However, the translocation is balanced in t(X;17) renal cell carcinoma and unbalanced in alveolar soft part sarcoma, which presumably explains the clinical and morphological differences. The function of chimeric TFE3 fusion proteins can also vary, which may explain the different histological features observed in this tumor entity of renal cell carcinoma.

### 1.1. Clinical Features

**Figure 1:**
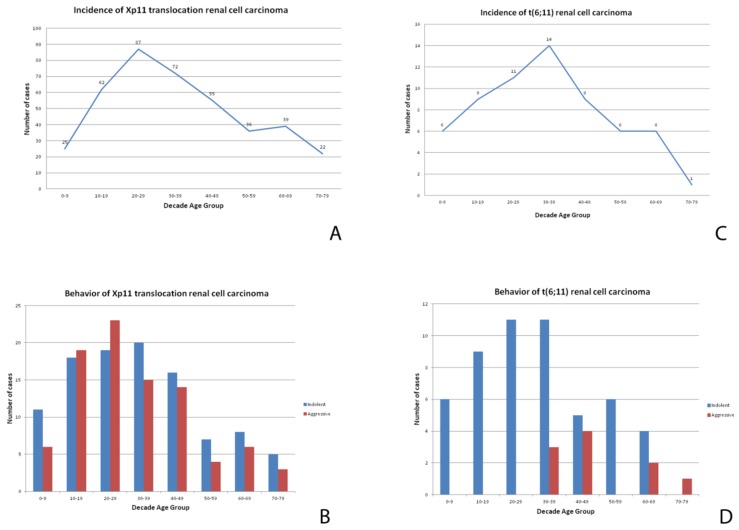
A chart showing the incidence (number of patients with tumors divided by the number of patients in the age group) of Xp11 translocation renal cell carcinomas (**A**) and t(6;11) renal cell carcinoma (**C**) at different ages. A chart showing the clinical behavior of Xp11 translocation renal cell carcinomas (**B**) and t(6;11) renal cell carcinoma (**D**) at different ages.

Xp11 translocation renal cell carcinoma comprises 20–75% of renal cell carcinomas in childhood [12] and 1–4% of adult renal cell carcinomas (calculated excluding patients younger than 18 years old) with an average age of onset of 40 years (Figure 1). The incidence of Xp11 translocation renal cell carcinoma in adults may be underestimated, likely for the morphological overlap with more common adult renal cell carcinoma subtypes, such as clear cell and papillary renal cell carcinoma. Considering an overall of 403 genetically confirmed Xp11 translocation renal cell carcinomas described in the literature, there is a slight female predominance (F:M ratio, 1.6:1). Clinically, there are no particular features typically presented. As other renal cell carcinomas, roughly one-third of all tumors are asymptomatic, often accidentally discovered. Prior exposure to cytotoxic chemotherapy has been reported as a risk factor [13]. 

### 1.2. Pathologic Features

#### 1.2.1. Gross Findings 

They usually present as solitary cortical masses characterized by tan-yellow cut surfaces with foci of hemorrhage and necrosis and occasionally focal cystic degeneration. Although there is no specific macroscopic appearance of Xp11 translocation renal cell carcinoma, they do not share the macroscopic features of clear cell renal cell carcinoma.

#### 1.2.2. Microscopic Features 

**Figure 2:**
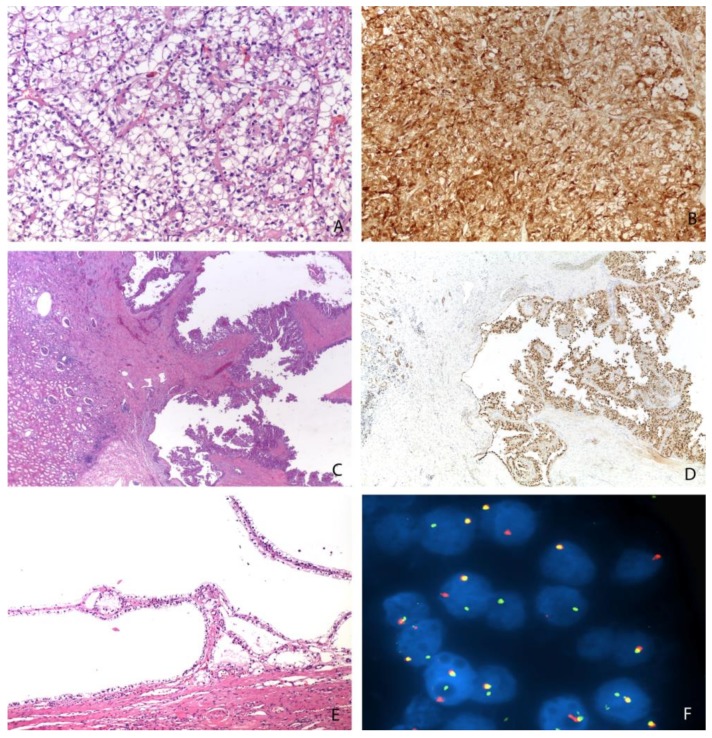
Different morphologies of Xp11 translocation renal cell carcinomas: resembling a clear cell renal cell carcinoma (Magnification: 200×) (**A**), showing a papillary (Magnification: 25×) (**C**) or cystic (Magnification: 100×) (**E**) pattern. An example of strong and diffuse expression of cathepsin K (Magnification: 200×) (**B**), the nuclear positivity of PAX8 (Magnification: 25×) (**D**), and the demonstration of *TFE3* gene rearrangement by FISH (Magnification: 1000×) (**F**).

Histologically, Xp11 translocation renal cell carcinomas are characterized by heterogeneous architectural and cytologic features, mimicking almost all subtypes of renal cell carcinoma [10,14]. The most distinctive morphologic pattern is the presence of a papillary architecture composed of epithelioid clear cells. However, different architectures have been reported, such as solid, nested, trabecular, and microcystic pattern. More frequently, tumor cells demonstrate voluminous clear to eosinophilic cytoplasm (Figure 2). The nuclei may show variability in size and are generally large with a prominent eosinophilic nucleolus (typically G3 by ISUP/WHO 2016) [1]. Psammoma bodies are often present. 

#### 1.2.3. Immunohistochemical Features and Fluorescent in Situ Hybridization (FISH) Analysis

Like other subtypes of renal cell carcinoma, Xp11 translocation renal cell carcinomas are positive for PAX8. Vimentin and cytokeratin 7 (CK7) are typically negative. Staining for CD10 and alpha-methylacyl-CoA racemase (AMACR) is generally reported. In one-third of all cases, Xp11 translocation renal cell carcinoma focally express melanogenic markers such as Melan-A and HMB45. Staining for cathepsin K is observed in a subset of Xp11 translocation renal cell carcinomas (approximately 50%) (Figure 2). Interestingly, PRCC-TFE3 renal cell carcinoma is labelled more frequently for cathepsin K than ASPL-TFE3 renal cell carcinoma [15,16]. TFE3 immunostaining, initially considered as the most sensitive and specific marker, should be cautiously used due to the not infrequent false-positive and false-negative results [17]. For this reason, the identification of the *TFE3* rearrangement by FISH assays on formalin-fixed and paraffin-embedded tissue sections is currently the gold standard to reach the correct diagnosis [17,18,19]. Of course, a reliable interpretation requires a univocal cut-off. However, different thresholds have been used to demonstrate the occurrence of translocation. A positive result was considered when >10%, >15%, or >20% of the neoplastic nuclei showed split signals. Nevertheless, an extensive review of previously reported cases of Xp11 translocation renal cell carcinoma in which it has been reported that the frequency of split signals in each case showed a high frequency of split signals (>40%). It is important to keep in mind that the FISH assay is unable to detect subtle *TFE3* gene inversions, such as those that result in the *RBM10-TFE3* gene fusion [20]. In the experience of the authors, minimally split fluorescent signals in which fluorescent signals were separated by a signal diameter or less were observed in few cases [21]. Recently, it has been argued whether *TFE3* gene rearrangement is the key event in tumorigenesis [22,23,24]. In our practice, the fraction of cells showing the translocation is commonly high, supporting the idea that it is the main driver event in tumorigenesis.

### 1.3. Differential Diagnosis 

Due to the wide spectrum of morphologies observed in Xp11 translocation renal cell carcinomas, the differential diagnosis is challenging, and it is important to consider these carcinomas in all unusual renal cell carcinomas occurring, especially in children and young adults [1]. Several neoplasms can be confused with Xp11 translocation renal cell carcinoma, mainly clear cell and papillary renal cell carcinomas. In this setting, cathepsin K is the most reliable immunohistochemical marker. Of note, as previously stated, immunolabelling for cathepsin K is observed in roughly half of all Xp11 translocation renal cell carcinomas. Other immunohistochemical markers may be helpful based on the differential diagnosis. CD10 is expressed in almost all Xp11 translocation renal cell carcinomas in analogy to clear cell renal cell carcinomas. However, carbonic anhydrase IX is usually negative or only focally present in Xp11 translocation renal cell carcinomas and positive in clear cell renal cell carcinomas, suggesting the usefulness of this marker in this particular differential diagnosis. On the other hand, AMACR (Alpha-methylacyl-CoA racemase) is frequently positive in Xp11 translocation renal cell carcinomas, as well as in papillary renal cell carcinomas, but CK7 is typically negative in Xp11 translocation renal cell carcinomas and positive in papillary renal cell carcinomas. Clear cell papillary renal cell carcinomas may be another tricky differential diagnosis [25]. Those tumors usually label for CK7 and GATA3, both not expressed in Xp11 translocation renal cell carcinomas. Finally, it is important to remember less frequent tumors, such as pure epithelioid PEComa/epithelioid angiomyolipoma. In those cases, PAX8 and CD68 (PG-M1) are extremely useful (see differential diagnosis of t(6;11) renal cell carcinomas).

### 1.4. Prognosis and Treatment 

The outcome of Xp11 translocation renal cell carcinoma is highly variable, from indolent to rapidly aggressive behavior [12,26,27]. Overall, Xp11 translocation renal cell carcinoma has a worse prognosis than papillary renal cell carcinoma and there is a similar prognosis for clear cell renal cell carcinoma [28]. Although several studies have claimed that Xp11 translocation renal cell carcinomas in children have a relatively indolent course, the review of the literature (Figure 1) shows a high percentage of aggressive cases in young adults. Among Xp11 translocation renal cell carcinoma, patients with ASPL-TFE3 fusion seem to have a worse prognosis and more frequently lymph node metastasis, but it is still unclear whether the fusion partner plays a prognostic role [28,29]. Considering an overall 403 genetically confirmed Xp11 translocation renal cell carcinomas described in the literature, 47% of cases (91 of 194 tumors with available follow up) behaved aggressively. When aggressive and non-aggressive cases are compared, we observe that recurrences or metastases occurred within 24 months from the surgery. It is worth noting that sarcomatoid or rhabdoid de-differentiation have never been reported. There is no statistical difference of age between aggressive and non-aggressive cases. As expected, a larger tumor size (*p* < 0.0001) correlates with aggressive behavior. Interestingly, the presence of necrosis, but not nucleolar grade, correlates with aggressiveness, the same prognostic characteristics reported in chromophobe renal cell carcinoma. 

With regard to the treatment, the optimal therapy for MiT family translocation renal cell carcinoma remains to be determined. For localized tumors, including patients with positive regional lymph nodes, surgery is the treatment of choice. For patients with hematogenous metastases, several attempts of therapy have been tried based on the treatment of clear cell renal cell carcinoma. Therapies targeting vascular endothelial growth factor receptor, immunotherapy, mTOR inhibitors, and target therapies for the MET signaling pathway are possible options [30,31,32,33,34]. Unfortunately, to date there is no data regarding predictive markers to choose the best therapy for an individual patient. In the past few years, the efficacy of Cabozantinib, a tyrosine kinase inhibitor with activity against c-MET, AXL, and vascular endothelial growth factor receptor 2, has been proven for the treatment of metastatic clear cell renal cell carcinomas [35,36] and recently for non-clear cell histologies [37]. Moreover, whole genome DNA and RNA sequencing studies have recently been reported on a small number of cases [10,38], providing the activity of other pathways which may present other potential targets for novel therapies [38].

## 2. t(6;11) Renal Cell Carcinoma

t(6;11) renal cell carcinoma is an extremely rare variant and accounts for 0.02% of all renal carcinomas. Although the initial description was in children [39], t(6,11) renal cell carcinoma may occur in adults. The t(6;11) translocation fuses the gene for *TFEB*, located on chromosome 6, with Alpha (*MALAT1*), a gene of unknown function, resulting in overexpression of TFEB.

### 2.1. Clinical Features

The t(6;11) renal cell carcinomas are less common than the Xp11 renal cell carcinomas; approximately 60 cases are documented in the literature, the majority of which in children and adolescents. However, it has been demonstrated that these neoplasms can occur in adults as well. The mean age of presentation is 34 years (Figure 1), with a wide reported range of 3–77 years. Conversely to Xp11 translocation renal cell carcinomas, in t(6;11) renal cell carcinoma there is no gender predominance (F:M ratio, 0.75:1). The tumor is usually an incidental finding. Similar to Xp11 translocation renal cell carcinoma, a subset of cases has occurred in patients who have received cytotoxic chemotherapy for other reasons. 

### 2.2. Pathologic Features

#### 2.2.1. Gross Findings

As Xp11 translocation renal cell carcinoma, t(6;11) renal cell carcinoma does not have a distinctive gross appearance.

#### 2.2.2. Microscopic Features

**Figure 3:**
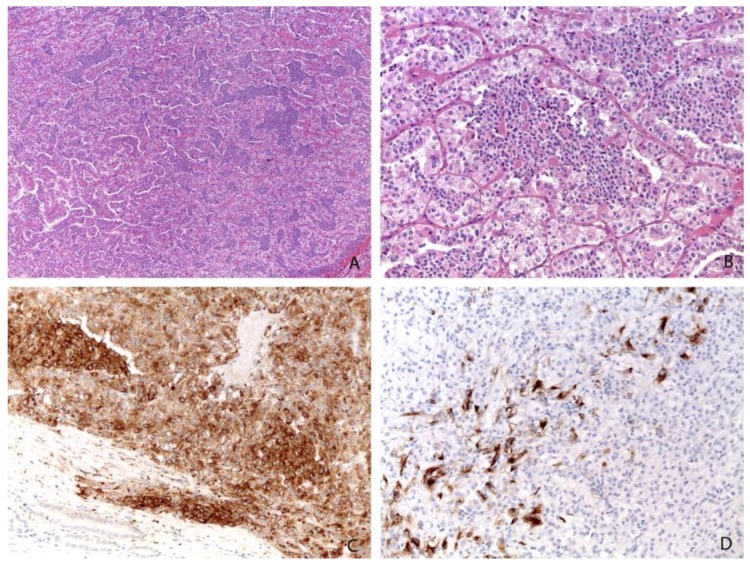
The most common morphology of t(6;11) renal cell carcinoma with larger epithelioid cells and smaller cells clustered around eosinophilic spheres formed by basement membrane material (**A**, Magnification: 25×; **B**, Magnification: 200×). Almost all cases are positive for cathepsin K (Magnification: 200×) (**C**) and HMB45 (Magnification: 200×) (**D**).

Histologically, t(6;11) renal cell carcinoma has been classically characterized by a distinctive biphasic morphology with larger epithelioid cells and smaller cells clustered around eosinophilic spheres formed by basement membrane material (Figure 3) [1,40]. However, several reports have shown a broad range of morphology in molecularly confirmed t(6;11) renal cell carcinomas [41]. Papillary and tubulocystic architectures, clear cell and oncocytoma-like features, and diffuse hyalinization with thick-walled blood vessels are some of the unusual pathological features described [1]. The cells typically show nucleolar grade G2 and G3 by ISUP/WHO 2016 [42].

#### 2.2.3. Immunohistochemical Features and FISH Analysis

Immunohistochemically, most t(6;11) renal cell carcinomas express PAX8, supporting renal tubular differentiation and melanogenesis markers, such as HMB-45 and Melan-A. Cathepsin K is overexpressed in almost all t(6;11) renal cell carcinomas [43,44]. Staining for TFEB was considered highly sensitive and specific for this tumor. However, the results can be inconsistent among laboratories, mainly because of technical factors such as fixation time and differences in the methods of antigen retrieval. Like Xp11 translocation renal cell carcinomas, the identification of the rearrangement by FISH analysis is the gold standard for the diagnosis [45]. As previously discussed for Xp11 translocation renal cell carcinomas, it is of paramount importance to define a proper cut-off to establish the occurrence of *TFEB* rearrangement, even in t(6;11) renal cell carcinoma, when the frequency of split signals is high (>38%). Although less frequently than in Xp11 translocation renal cell carcinoma, we observed minimally split fluorescent signals. 

### 2.3. Differential Diagnosis 

The wide spectrum of morphology results in several differential diagnoses including Xp11 translocation renal cell carcinoma, pure epithelioid PEComa/epithelioid angiomyolipoma, and other more common types of renal cell carcinoma [46,47,48]. Among them, pure epithelioid PEComa/epithelioid angiomyolipoma is the most challenging diagnosis in clinical practice [42]. Indeed, the two entities share the immunohistochemical expression of melanogenesis markers and cathepsin K, and both are often negative for cytokeratin. PAX8 immunoreactivity and CD68 (PG-M1) negativity supports the diagnosis of t(6;11) renal cell carcinoma, whereas pure epithelioid PEComa/epithelioid angiomyolipoma is PAX8 negative and CD68 (PG-M1) positive [42].

### 2.4. Prognosis and Treatment 

Most instances of t(6;11) renal cell carcinoma have an indolent clinical course. An aggressive behavior is observed in roughly 17% of the cases (11 of 64 tumors with available follow up). Larger masses (*p* = 0.04) and older patients (*p* = 0.007) seem to be parameters correlated with aggressiveness. It should be noted that hematogenous metastases seem to be more common than nodal metastases. To date, there are no well-established prognostic markers to predict the biological behavior. However, it is possible that an increase in the copy number of the *TFEB* gene region in t(6;11) renal cell carcinoma may predict an aggressive clinical course [42,49]. The radical surgery remains the best therapeutic strategy. Because of the rarity of this tumor, no information regarding neoadjuvant or adjuvant therapies are available. Since these neoplasms have demonstrated the capacity to recur, follow-up examinations are important for these patients.

## 3. Renal Cell Carcinoma with *TFEB* Amplification

**Figure 4:**
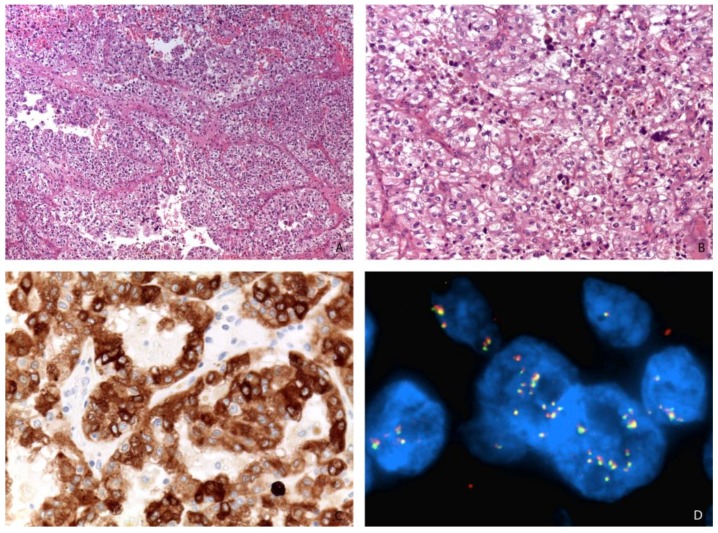
A high-grade renal cell carcinoma (**A**, Magnification: 50×; **B**, Magnification: 200×) expressing Melan-A (Magnification: 400×) (**C**) and showing *TFEB* gene amplification by FISH (Magnification: 1000×) (**D**).

More recently, renal cell carcinomas with *TFEB* amplification have been described and appear to be associated with a poor outcome [50,51,52,53,54,55]. *TFEB* amplification in renal cell carcinoma can occur independently of or in association with *TFEB* rearrangement [50,55]. *TFEB* gene rearrangement or amplification increases TFEB expression which causes the subsequent expression of immunohistochemical markers such as cathepsin K, Melan-A, and HMB45 [43]. Nevertheless, *TFEB*-amplified renal cell carcinomas are different from t(6;11) renal cell carcinomas [50]. First, they typically occur in older patients (mean 65 years) compared to unamplified t(6;11) renal cell carcinoma (mean age 34 years). Second, their morphology is usually high grade and less typical than the biphasic appearance of t(6;11) renal cell carcinoma (Figure 4). Third, melanogenic marker expression is less reliable: while all cases have expressed Melan-A, roughly half of the cases express cathepsin K and HMB45, usually positive in t(6;11) renal cell carcinoma. Fourth, TFEB amplified renal cell carcinomas typically have a poor outcome while t(6;11) renal cell carcinomas are usually indolent. Of note, it has been demonstrated that renal cell carcinomas showing *TFEB* amplification harbor concurrent vascular endothelial growth factor A (*VEGFA*) gene amplification [52,55]. This is may be due to the proximity of those two genes, which are both located on the short arm of chromosome 6. With regard to the treatment, Gupta et al. hypothesized the possible usefulness of VEGFR-targeted therapy in a few cases of renal cell carcinomas with *TFEB/VEGFA* coamplification [52].

## 4. Comparison of Xp11 Translocation Renal Cell Carcinoma and t(6;11) Renal Cell Carcinoma

As illustrated in Table 1, Xp11 translocation renal cell carcinoma and t(6;11) renal cell carcinoma differ in several ways. Xp11 translocation renal cell carcinoma seems to occur in patients younger than t(6;11) renal cell carcinoma; with a slight female predominance and a more frequently aggressive clinical course. Conversely to Xp11 translocation renal cell carcinoma, the immunohistochemical analysis of t(6;11) renal cell carcinoma is more consistent, showing the overexpression of cathepsin K and melanogenesis makers in almost all cases.

## 5. Conclusions

On the basis of their clinical, immunohistochemical, and molecular similarities, the last WHO classification grouped Xp11 translocation renal cell carcinoma and t(6;11) renal cell carcinoma together under the name “MiT family translocation renal cell carcinoma”. However, among them there are few differences, mainly in morphology and clinical behavior. For those reasons, we suggest to keep the distinction in the clinical practice. Overall, this review emphasizes that MiT family translocation renal cell carcinoma is a distinctive entity and therefore stresses the importance of recognizing it as a specific category of renal cell carcinoma to properly identify these cases in future clinical trials looking for effective therapies.

## Figures and Tables

**Table 1:** Main differences between Xp11 translocation renal cell carcinoma and t(6;11) renal cell carcinoma.

Parameter	Xp11 Translocation RCC	t(6;11) RCC
Clinical		
Age distribution	peak: 20–29 years	peak: 30–39 years
Gender	F:M ratio, 1.6:1	F:M ratio, 0.75:1
Behavior	aggressive in 47% of cases	aggressive in 17% of cases
Morphology		
features	broad spectrum	usually biphasic
Immunohistochemistry		
Cathepsin K	47% positive	94% positive
Melan-A	39% positive	91% positive
HMB45	32% positive	83% positive

RCC: renal cell carcinoma; F: female; M: male.
